# Short-Term Effects of Air Pollution on Cardiovascular Hospitalizations in the Pisan Longitudinal Study

**DOI:** 10.3390/ijerph18031164

**Published:** 2021-01-28

**Authors:** Salvatore Fasola, Sara Maio, Sandra Baldacci, Stefania La Grutta, Giuliana Ferrante, Francesco Forastiere, Massimo Stafoggia, Claudio Gariazzo, Camillo Silibello, Giuseppe Carlino, Giovanni Viegi, on behalf of the BEEP Collaborative Group

**Affiliations:** 1 Institute for Biomedical Research and Innovation, National Research Council, 90146 Palermo, Italy; stefania.lagrutta@irib.cnr.it (S.L.G.); fran.forastiere@gmail.com (F.F.); giovanni.viegi@irib.cnr.it (G.V.); 2 Institute of Clinical Physiology, National Research Council, 56126 Pisa, Italy; saramaio@ifc.cnr.it (S.M.); baldas@ifc.cnr.it (S.B.); 3 Department of Health Promotion, Mother and Child Care, Internal Medicine and Medical Specialties, University of Palermo, 90127 Palermo, Italy; giuliana.ferrante@unipa.it; 4 Department of Epidemiology, Lazio Region Health Service—ASL Roma 1, 00147 Rome, Italy; m.stafoggia@deplazio.it; 5 Occupational and Environmental Medicine, Epidemiology and Hygiene Department, Italian Workers’ Compensation Authority (INAIL), Monte Porzio Catone, 00144 Rome, Italy; c.gariazzo@inail.it; 6 Arianet S.r.l., 20128 Milan, Italy; c.silibello@aria-net.it; 7 Simularia S.r.l., 10121 Turin, Italy; g.carlino@simularia.it

**Keywords:** air pollution, cardiovascular hospitalizations, case-crossover design, epidemiology, high-resolution pollutant estimates, small cities, suburban areas

## Abstract

Air pollution effects on cardiovascular hospitalizations in small urban/suburban areas have been scantly investigated. Such effects were assessed among the participants in the analytical epidemiological survey carried out in Pisa and Cascina, Tuscany, Italy (2009–2011). Cardiovascular hospitalizations from 1585 subjects were followed up (2011–2015). Daily mean pollutant concentrations were estimated through random forests at 1 km (particulate matter: PM_10_, 2011–2015; PM_2.5_, 2013–2015) and 200 m (PM_10_, PM_2.5_, NO_2_, O_3_, 2013–2015) resolutions. Exposure effects were estimated using the case-crossover design and conditional logistic regression (odds ratio—OR—and 95% confidence interval—CI—for 10 μg/m^3^ increase; lag 0–6). During the period 2011–2015 (137 hospitalizations), a significant effect at lag 0 was observed for PM_10_ (OR = 1.137, CI: 1.023–1.264) at 1 km resolution. During the period 2013–2015 (69 hospitalizations), significant effects at lag 0 were observed for PM_10_ (OR = 1.268, CI: 1.085–1.483) and PM_2.5_ (OR = 1.273, CI: 1.053–1.540) at 1 km resolution, as well as for PM_10_ (OR = 1.365, CI: 1.103–1.690), PM_2.5_ (OR = 1.264, CI: 1.006–1.589) and NO_2_ (OR = 1.477, CI: 1.058–2.061) at 200 m resolution; significant effects were observed up to lag 2. Larger ORs were observed in males and in subjects reporting pre-existent cardiovascular/respiratory diseases. Combining analytical and routine epidemiological data with high-resolution pollutant estimates provides new insights on acute cardiovascular effects in the general population and in potentially susceptible subgroups living in small urban/suburban areas.

## 1. Introduction

The burden of air pollution in terms of cardiovascular diseases (CVD) has been thoroughly documented [1]. Ambient air pollution accounts for 25% of disability-adjusted life years lost for stroke and 23% for ischemic heart disease worldwide, with the highest burden affecting elderly people [2]. Recently, the US Environmental Protection Agency has stated that there is a causal relationship between fine particulate matter (PM) exposure (PM with aerodynamic diameter ≤2.5 microns, i.e., PM_2.5_) and cardiovascular events (emergency room visits, acute hospital admissions, and mortality) observed from hours to days after the exposure [3]. In a US study across the Mid-Atlantic, a short-term increase of 10 μg/m^3^ in PM_2.5_ was associated with a 0.78% increase in CVD admission rate in elderly people [4]. Exposure to nitrogen dioxide (NO_2_) was also found to be a risk factor for myocardial infarction (MI) hospitalization (29% higher risk for a 10 μg/m^3^ increase) in three industrial cities in southern Poland [5].

Despite the largely available evidence regarding the effects of air pollution on CVD hospitalizations, such effects have been scantly investigated in small urban and suburban areas. Indeed, most of the previously published studies were carried out in large (e.g., metropolitan) and/or highly polluted (e.g., industrial) areas [5,6,7,8,9], since a large amount of observed data are available from monitoring stations in such contexts. Consequently, generalizability may be limited and a selection bias may occur [4]. Indeed, previous studies focusing on medium-sized or lowly polluted areas considered mean concentration levels recorded by a few (1 or 2) monitoring stations for assessing the health effects [10,11]. More in general, the assessment of individual exposures is often derived through averaged values across the monitoring stations at city level or zip-code level [4,12], leading to a possible underestimation of the exposure–outcome relationship [13]. Moreover, time-series or other aggregate study designs have limitations in the ability to assess potential individual effect modifiers, such as smoking habits and occupational exposure [14]. Conversely, the use of data from analytical epidemiological surveys let us overcome these limitations.

The “Big data in Environmental and occupational EPidemiology” (BEEP) project aimed to assess the health effects of air pollution considering the whole Italian territory, including specific urban, suburban and rural areas, and using measures of air pollutant exposure estimated at residential level, with a resolution of 1 km [15,16,17] and 200 m [18].

The aim of this study was to assess the risk of hospitalization for CVD among the participants in the analytical epidemiological survey carried out during the period 2009–2011 in a small urban (Pisa) and a suburban (Cascina) area of Tuscany (Italy). We used high-resolution estimates of PM_10_ (PM with aerodynamic diameter ≤ 10 microns), PM_2.5_, NO_2_, and O_3_ (ozone) available from the BEEP project.

## 2. Materials and Methods

### 2.1. Study Design and Area

**Figure 1:**
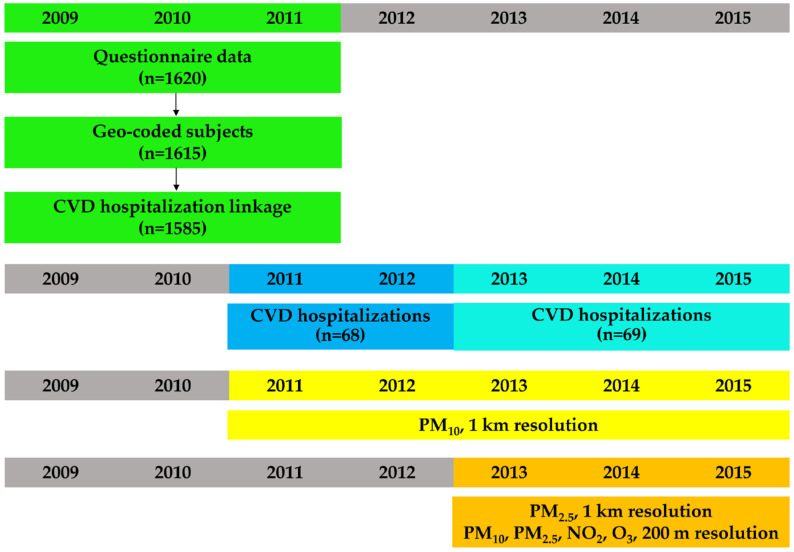
Data collection timeline.

This case-crossover study was applied to the data collected during the third analytical cross-sectional survey (2009–2011) performed in Pisa and Cascina by the Pulmonary Environmental Epidemiology Unit of the Institute of Clinical Physiology of the Italian National Research Council (CNR) [19,20,21]. Within the framework of the BEEP project, 1615 of the 1620 participants were geocoded and linked to estimated levels of air pollution: of them, 1585 (98%) were followed-up during the period 2011–2015 and linked to a hospital discharge database (Figure 1).

The Pisa University Hospital Ethics Committee provided approvals for the 2009–2011 survey protocol, participant information sheet, and consent form (Prot. No. 23,887, 16 April 2008) and for using individual data in the statistical analyses of this manuscript (Prot. No. 24,567, 8 May 2018).

At the time of the study, the province of Pisa was subdivided into 39 municipalities, with a total area of 2448 km^2^, about 410,000 inhabitants and an average density of 166 inhabitants/km^2^. Over half of the total population was living in just five municipalities, which altogether reached over than 200,000 inhabitants and represented the most densely populated areas: Pisa, Cascina, San Giuliano Terme, Pontedera, and San Miniato.

**Figure 2:**
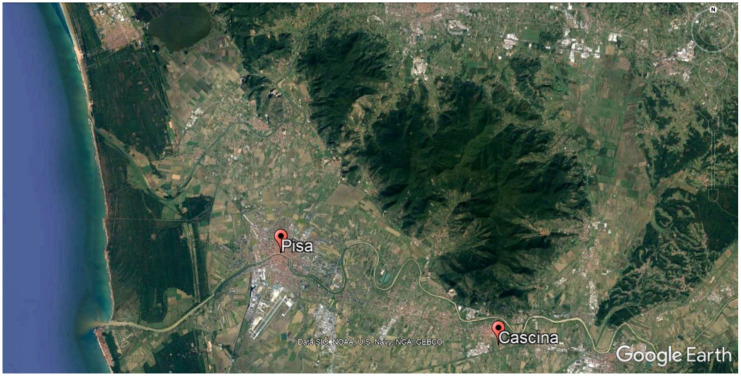
Map of the study area (1 cm = 2 km).

Pisa is a small urban area (185 km^2^) located a few kilometers from the mouth of the Arno River (43°43′ N 10°24′ E), in a flat area surrounded to the East by the Pisan mountain (maximum altitude 917 m) (Figure 2). The average resident population in 2009–2011 consisted of 87,058 individuals.

Pisa is characterized by residential areas and urban and inter-urban roads. In 2009–2010, data from fixed monitoring stations provided by the Tuscany Environmental Protection Agency showed a mean annual concentration of 31 µg/m^3^ for PM_10_ (below the EU accepted limit: 40 µg/m^3^), 16 µg/m^3^ for PM_2.5_ (below the EU accepted limit: 25 µg/m^3^) and 27 µg/m^3^ for NO_2_ (below the EU accepted limit: 40 µg/m^3^).

Cascina is a suburban area (79 km^2^) located on the left bank of the Arno river, in its alluvial plain at 6 m above sea level, 16 km South-East of Pisa (43°40′ N 10°30′ E) (Figure 2). The average population in 2009–2011 consisted of 43,878 residents. The municipality is characterized by agricultural and handcraft activities, sparse buildings and intersections with very small streets. The main source of vehicular traffic pollution is a highway connecting Pisa to Florence, crossing Cascina and entering the easternmost part of Pisa. Fixed monitoring stations are no longer present in the area since 2011. In 2009–2010, data provided by the Tuscany Environmental Protection Agency showed mean annual concentrations of 33 µg/m^3^ for PM_10_ (below the EU accepted limit: 40 µg/m^3^) and 27 µg/m^3^ for NO_2_ (below the EU accepted limit: 40 µg/m^3^); PM_2.5_ was not monitored.

In the study area, historical meteorological variables collected by Pisa International Airport indicate a prevailing wind direction from the East between September and April, and from the West between May and August.

### 2.2. Questionnaire Data

Information about individual risk factors was collected during the 2009–2011 survey through a standardized interviewer-administered questionnaire [19,20,22]. The area of residence was defined as urban area (Pisa) or suburban area (Cascina). Age was calculated in years from birth and questionnaire compilation dates, and it was categorized as <85 or ≥85 years (approximately the median age of the subjects hospitalized during the period 2013–2015). Gender was defined as male or female. Smoking status was split into non-smokers (subjects who had never smoked for at least one year) or ever smokers (subjects who currently smoked at least one cigarette daily or subjects who had quit smoking before the survey and did not smoke at the time of the survey). Occupational exposure was considered in case of exposure to at least one among fumes, gases, dusts and chemicals in the working environment during the lifetime. Pre-existent cardiovascular/respiratory diseases were defined as a reported physician diagnosis of heart attack, heart failure, asthma or chronic obstructive pulmonary disease.

### 2.3. Cardiovascular Hospitalizations

Data from hospital discharge records were linked to 1585 geo-coded subjects for the years 2011–2015, when environmental data were available. Each record included information about the date of hospital admission (used to calculate the age at hospitalization), primary diagnosis and type of admission. Only acute (unscheduled) hospitalizations with a CVD as the primary cause (International Classification of Diseases, 9th Revision—ICD9: 390–459) were considered in this study.

### 2.4. PM_10_, and PM_2.5_, 1 km Resolution

Daily mean concentrations of PM_10_ at 1 km resolution were estimated for the years 2011–2015 through a random forest machine learning approach (RFMLA), while models for PM_2.5_ were restricted to 2013–2015, since the availability of PM_2.5_ monitors before 2013 was very limited [15,23]. For each day and each squared kilometer of Italy, several spatiotemporal parameters were collected, such as PM monitored data from the available monitoring sites, resident population, road density data, satellite-based aerosol optical depth (AOD) data, and land use and meteorological data. A four-stage model was therefore trained to predict daily PM_10_ and PM_2.5_ concentrations for each 1 × 1 km^2^ grid cell, using the aforementioned spatiotemporal parameters as predictors of PM_10_ and PM_2.5_ monitoring data (target variables). The whole estimation process is fully described elsewhere [15,23].

Focusing on our study area, cross-validated (10-fold) R^2^ was 0.75 (2011–2015) and 0.84 (2013–2015) for PM_10_, and 0.86 (2013–2015) for PM_2.5_. Such R^2^ was calculated considering the Pisan background monitoring station of Passi (43°44′16″ N 10°24′02″ E), providing data for all the pollutants. The daily series of exposure levels estimated on the grid cells was linked to the residential addresses of the subjects according to their spatial locations.

### 2.5. PM_10_, PM_2.5_, NO_2_ and O_3_, 200 m Resolution

Daily mean concentrations of air pollutants for the years 2013–2015 at the resolution of 200 m were estimated using an RFMLA as well. The spatiotemporal predictors were the concentration fields computed by a flexible air quality regional (FARM) model, the Normalized Difference Vegetation Index (NDVI), Julian day, day of week and month, resident population, mean elevation and daily traffic volumes [18]. The RFMLA model was then applied in two phases: a tuning (or training) phase in which the monitoring sites were used for choosing the best set of model predictors, and a generalization phase aimed to estimate the concentrations elsewhere. The whole estimation process is fully described in another publication of the BEEP project [18].

Focusing on our study area (Passi monitor), cross-validated (10-fold) R^2^ was 0.82 for PM_10_, 0.78 for PM_2.5_, 0.71 for NO_2_, and 0.71 for O_3_. The daily series of exposure levels estimated on the grid cells were linked to the residential addresses of the subjects according to their spatial locations.

### 2.6. Time-Varying Confounders

A summer population reduction was defined as a categorical variable assuming value “1” from mid-July to end-August and “0” otherwise. Holidays were coded as a categorical variable assuming value “1” at Christmas and Easter (and days around) and other isolated national holidays, and “0” during normal days. Influenza epidemics data were retrieved from the Italian Institute of Health (Istituto Superiore di Sanità) and were defined as a categorical variable assuming value “1” in the periods with the highest incidence and “0” otherwise. Daily means of air apparent temperature were retrieved by the European Centre for Medium-Range Weather Forecasts Re-Analysis (ERA)–Interim project, at the spatial resolution of 10 km [24].

### 2.7. Statistical Analyses

The association between air pollution levels and the risk of CVD hospitalizations was investigated using a case-crossover design [25]. A symmetrical and bidirectional selection of control days was performed to adjust for time trends (day of the week, long-term and seasonal trends) and slowly varying or time-invariant covariates: one control day at a fixed interval of 7 days before the episode (day −7), and another one 7 days after the episode (day +7) [26].

For CVD hospitalizations, an explorative analysis was performed by reporting their spatiotemporal distribution and risk factor stratification.

For all the study days (case and control days pooled), we reported the mean, standard deviation (SD), median, 25th and 75th percentile, and the interquartile range (IQR) of the estimated pollutant levels. Mean (SD) estimated pollutant concentrations were compared between case days (day 0) and control days (day −7 and day +7) through paired *t*-tests.

Conditional logistic regression models with distributed lags were estimated for each pollutant. We first considered all the cases (non-stratified analysis), and then stratified by risk factor categories. Lagged effects were estimated up to the 6th day before the episode. Due to the high collinearity of between-day concentrations, and the limited sample size, the lagged effects were constrained through linear functions [12]: a linear function in the lag, and a linear function in the lag logarithm. The best lag structure was selected based on the Bayesian information criterion (BIC) (the lower the better) [27].

All the estimated effects were adjusted for population decreases during summer vacation periods, holidays, influenza epidemics, and apparent temperature. The effect of temperature was controlled for by calculating the average of current- and previous-day apparent temperature (lag 0–1) and using linear and quadratic terms [28]. However, an exploratory analysis showed that no effects of the aforementioned confounders were statistically significant, and the adjusted models had rather worse BICs compared to unadjusted models. Therefore, also considering the small sample size, the confounders were not included in the final analyses, in order to obtain parsimonious models and, consequently, to improve the stability of the estimated pollutant effects.

In the non-stratified analysis, pollutant effects were expressed as odds ratios (ORs) per 10 μg/m^3^ increase, and 95% confidence intervals (CIs). The effects of PM_10_ concentrations at 1 km resolution were estimated in both the period 2011–2015 and the sub-period 2013–2015, for the sake of comparability with the effects of the other pollutant estimates.

Sensitivity analyses were run using a “time-stratified” design [26] for the selection of control days (using the same days of the week within each month), and considering observed (Passi monitor) pollution levels (varying between days but not between subjects). In the stratified analyses, the effects were expressed as log-ORs and were visually displayed (only for the sub-period 2013–2015).

A formal test of effect modification was performed for each risk factor (e.g., males vs. females) by setting up a unique model with two interaction terms. The first interaction term accounted for the change in the intercept of the linear lag function, while the second one accounted for the change in the slope. The significance of the interactions was assessed through a likelihood-ratio test comparing the full model (with interactions) and the reduced model (without interactions).

All the statistical analyses were performed through R version 3.6.3 (R Foundation for Statistical Computing, Vienna, Austria). Statistical significance was set at *p* < 0.05.

## 3. Results

The mean age of the whole cohort (n = 1585) was 56.7 years (range: 18–103; SD = 18.2); 645 subjects (41%) were living in the urban area, 940 (59%) in the suburban area (Table S1). A total of 137 CVD hospitalizations occurred during the whole study period (2011–2015, Table 1): of these, 69 (50%) occurred during the sub-period 2013–2015 (Table S2), when the exposure estimates were available for all the pollutants. The mean age of the subjects hospitalized during the period 2011–2015 was 79.8 years (range: 32–94; SD = 10.6): 50 hospitalizations (36%) concerned subjects aged ≥85 years; 55 cases (40%) were observed in the urban area, 82 (60%) in the suburban area (Table 1). The mean age of the subjects hospitalized during the period 2013–2015 was 81.0 years (range: 32–94; SD = 10.7): 32 hospitalizations (46%) concerned subjects aged ≥85 years; 28 cases (41%) were observed in the urban area, 41 (59%) in the suburban area (Table S2).

Throughout all the study days (case and control days pooled), the IQR was approximately equal to 10 µg/m^3^ for all the pollutants, except for O_3_ which showed an IQR of about 30 µg/m^3^ (Table 2). Table S3 summarizes the mean (SD) of estimated pollution levels by year and area. Estimated pollution levels on the case days were higher than on the control days (except for O_3_): significant differences were found for PM_10_ (Table 3).

### Acute Effects of Air Pollutants on CVD Hospitalizations

Based on the BIC, unadjusted models with a linear function in the lag yielded the best trade-off between goodness-of-fit and model parsimony. During the period 2011–2015 (n = 137), significant effects were found for PM_10_ (1 km resolution) at lag 0 (OR = 1.137, 95% CI: 1.023–1.264) and up to lag 2 (Table 4). During the period 2013–2015 (n = 69), significant effects at lag 0 were found for PM_10_ (OR = 1.268, 95% CI: 1.085–1.483) and PM_2.5_ (OR = 1.273, 95% CI: 1.053–1.540) at 1 km resolution, as well as for PM_10_ (OR = 1.365, 95% CI: 1.103–1.690), PM_2.5_ (OR = 1.264, 95% CI: 1.006–1.589) and NO_2_ (OR = 1.477, 95% CI: 1.058–2.061) at 200 m resolution (Table 4). For PM and NO_2_, all the effects up to lag 2 were significant (decreasing with the lag). Conversely, no significant association was found for O_3_.

In the sensitivity analyses, similar results were found when using the time-stratified design for the selection of control days (Table S4). When considering the observed (Passi monitor) pollution levels, PM effects were similar to those estimated at the resolution of 1 km, whilst tendentially lower than those estimated at the resolution of 200 m (Table S5). Larger differences emerged for NO_2_.

**Figure 3:**
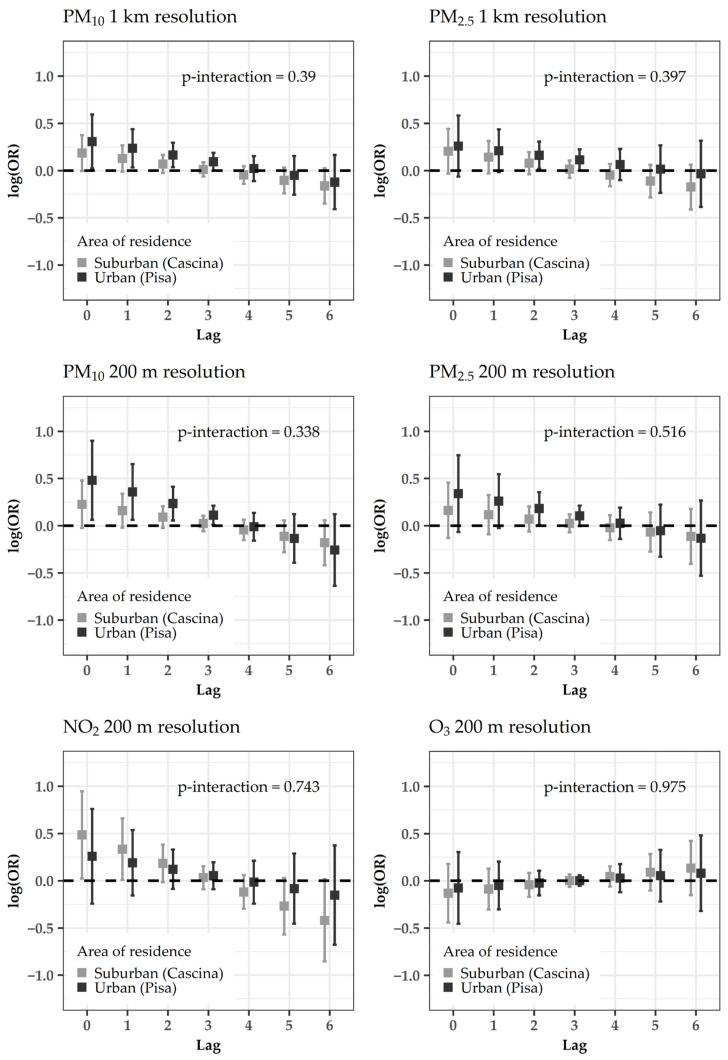
Acute effects of estimated pollution levels (sub-period 2013–2015) on the risk of cardiovascular hospitalizations: log-odds ratios (OR) and 95% confidence intervals for the conditional logistic regression models with distributed lags, stratified by area of residence. *p*-values for interactions are superimposed.

**Figure 4:**
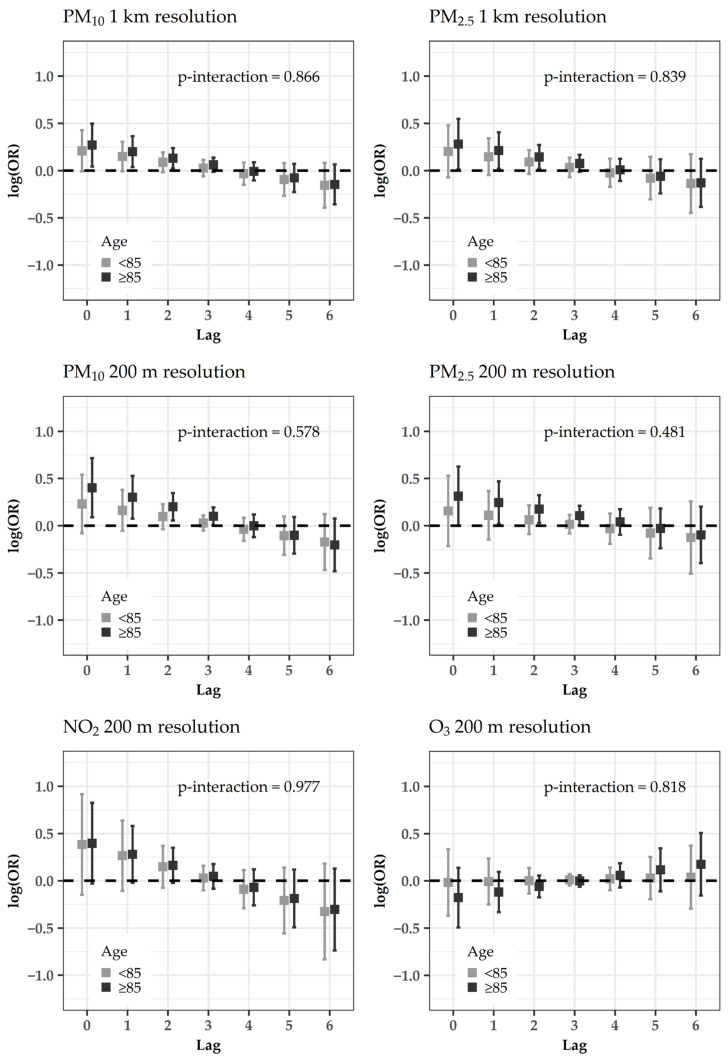
Acute effects of estimated pollution levels (sub-period 2013–2015) on the risk of cardiovascular hospitalizations: log-odds ratios (OR) and 95% confidence intervals for the conditional logistic regression models with distributed lags, stratified by age group. *p*-values for interactions are superimposed.

In stratified models, PM effects tended to be larger for the urban area (Figure 3), people aged ≥85 years (Figure 4), males (Figure S1), ever smokers (Figure S2), subjects reporting occupational exposures (Figure S3) and pre-existent cardiovascular/respiratory diseases (Figure S4). The interaction effect was statistically significant between gender and PM_2.5_ at the resolution of 200 m (p-interaction = 0.043, Figure S1), and between cardiovascular/respiratory disease pre-existence and PM_10_ at the resolution of 200 m (p-interaction = 0.049, Figure S4). A slightly larger effect of NO_2_ was evident for the suburban area (Figure 3).

## 4. Discussion

This study provides new insights into the relationship between short-term exposure to air pollutants and CVD hospitalizations in small urban/suburban areas, combining analytical epidemiological data with high-resolution pollutant estimates. Slightly larger effects were obtained when using exposure levels estimated at the individual residential addresses compared to monitor levels, and when considering potentially more susceptible subgroups. Moreover, concerning the exposure levels estimated at the individual residential addresses, a slightly larger effect was detected for PM_10_ at 200 m resolution compared to 1 km resolution; this did not occur for PM_2.5_. However, the small number of cases, especially in the sub-period 2013–2015 (for which exposure at 200 m resolution was available), may have led to less precise and slightly overestimated health effects. It is important to point out that, since the identification of the models stems from variation in pollution levels, such variation was assessed through some explorative analyses, reported in Table 2 and Table S3. Though the study areas are relatively small, the estimated pollution levels exhibited a fair variability overall (IQR ≈ 10 µg/m^3^), over time and across areas (Table S3). Indeed, although the estimated pollution levels were, on average, below the EU accepted limits, they tended to be higher in the case days with respect to the control days (Table 3).

### 4.1. Acute Effects of PM on CVD Hospitalizations

The effect sizes reported in our study (Table 4) for PM_10_ at 1 km resolution for 2011–2015 are similar to those of a recent case-crossover study carried out in the medium-polluted province of Quang Ninh (Vietnam, mean PM_10_ concentration 39.5 μg/m^3^, mean PM_2.5_ concentration 26.8 μg/m^3^). In such a study, the risk of CVD hospitalization among adults (age 15+) increased by about 15% for an IQR increase in PM during the period 2014–2016 [10]. Similar results were also found in a time series (2010–2011) study on elderly (≥60 years) living in a Brazilian, lowly polluted (mean PM_10_ concentration 12.7 μg/m^3^, mean PM_2.5_ concentration 4.4 μg/m^3^), medium-sized urban area. In such a study, the risk of CVD hospitalization increased by 19.6% for a 10 μg/m^3^ increase in PM_2.5_ (lag 0) [11]. Moreover, a recent case-crossover study (2014–2015) in highly polluted small/medium-sized industrial cities in southern Poland, reported a 16% increased risk of hospitalization for acute myocardial infarction per 10 μg/m^3^ increase in PM_10_, and a 20% increased risk for PM_2.5_ (lag 0) [5].

Time-series studies performed in Mediterranean areas reported much weaker associations. A 12.4 μg/m^3^ (IQR) increase in PM_2.5_ (lag 2–5 days) was associated with a 1.8% increased risk of all-age CVD hospitalizations (2011–2014) in five medium-large urban areas located in Central and eastern Europe characterized by low levels of air pollution (Augsburg, Dresden, Ljubljana, Chernivtsi and Prague) [6]. In ten urban areas of southern Europe characterized by medium-high levels of air pollution (Milan, Turin, Bologna, Parma, Reggio Emilia, Modena, Rome, Marseille, Madrid and Barcelona), 10 μg/m^3^ increases in PM_10_ and PM_2.5_ (lag 0–1 days) were associated, respectively, with a 0.53% and 0.51% increased risk of CVD hospitalizations among adults (age 15+, 2001–2010) [7]. The large effects reported in our study for PM_10_ at 1 km resolution for 2011–2015 (from 13.7% at lag 0 to 6.2% at lag 2) may be ascribed to a lower precision due to the much smaller sample size. On the other side, the small effects reported in the previous studies might also be ascribed, in part, to the use of a large-scale time-series study design, where proxies of individual exposure were derived by averaging measurements across the city monitoring stations, which may be located at a large distance from the resident houses [8,29]. Of note, in our study, the health effects of pollution levels estimated for 2013–2015 at the resolution of 200 m were somewhat larger than those obtained using pollution levels observed at the monitoring station (Table S5).

The need for improving exposure assessment has been highlighted in recent studies [4,12]. In particular, the use of spatiotemporal prediction models may reduce the risk of exposure misclassification arising from using a single value of daily exposure through the whole metropolitan area [4]. A previous case-crossover study assessed the effect of PM_2.5_ using an AOD-based exposure model at 10 km spatial resolution in lowly polluted urban and rural areas of Mid-Atlantic (maximum PM_2.5_ concentration 18.31 μg/m^3^). This study reported a 0.78% increase in CVD admission rate in elderly people (age 65+) for every 10 μg/m^3^ increase in PM_2.5_ (lag 0–1, period 2000–2006) [4]. More recently, PM_2.5_ levels were estimated at 1 km spatial resolution through a neural network combining satellite remote censoring data, chemical transport models, land use, and meteorology in the lowly polluted US New England region. This study reported an increase of about 1% in the risk of CVD hospitalizations (2000–2012) in elderly people (age 65+) for each 10 μg/m^3^ increase in PM_2.5_ up to lag 2 [12].

In both the aforementioned studies, differently from ours, the pollution values were linked to the zip codes of the cases rather than to their residential addresses, and the spatial resolution of 200 m was not attained in the exposure assessment. Indeed, the resolution of 1 km may still disregard local-scale phenomena, such as the low dispersion conditions determined by the city’s structure (e.g., street canyons and larger surface roughness caused by the presence of buildings, etc.), which might cause accumulation of pollutants [18,30]. In our study, we found that the effect of PM_10_ (2013–2015) at the resolution of 200 m was somewhat larger than the effect of the same pollutant at the resolution of 1 km, while the effect of PM_2.5_ was similar for both resolutions. This would be in agreement with the more ubiquitous nature of PM_2.5_ (mainly composed of secondary components) compared to PM_10_, due to a relatively lower deposition velocity [31].

The use of a fine spatial resolution would be particularly crucial in the estimation of NO_2_ concentrations, due to the high spatial variation, mainly explained by traffic [32]. In our study, NO_2_ exposure yielded the largest associations with CVD hospitalization for 2013–2015. Larger effects of NO_2_ compared to PM were also found for MI hospitalizations (2014–2015) in the three Polish industrial areas, showing about 29% higher risk (for a 10 μg/m^3^ increase) at lag 0, and 26% higher risk at lag 1 [5]. In an Iranian study (2010–2012) carried out in an industrial urban area, a 10 μg/m^3^ increase in NO_2_ was associated with a 26% higher risk of all-age hospitalization for atrial fibrillation after 24 h, while no significant associations emerged for PM exposure [33]. National data, from England and Wales (2003–2008) also highlighted a significant association with all-age CVD hospitalizations for NO_2_ but not for PM [34].

In our study, O_3_ exposure at the resolution of 200 m (2013–2015) was not significantly associated with CVD hospitalization, in line with several other studies carried out in urban/industrial areas of Italy, Iran, Taiwan and USA [9,12,33,35,36]. Indeed, epidemiological studies have strengthened the evidence that daily exposure to high levels of O_3_ increases cardiovascular mortality, while contrasting findings have been reported for CVD hospital admissions [37]. In Tuscany, a time series and case-crossover study carried out in five urban areas (2002–2005), including Pisa, showed an adverse effect of O_3_ exposure on out-of-hospital coronary deaths, but not on hospitalized acute MI [37]. This result was confirmed by another case-crossover study performed in Stockholm (2000–2010), where O_3_ exposure was associated with an increased risk of out-of-hospital cardiac arrest [38]. These findings may suggest that coronary deaths potentially related to increased levels of O_3_ occur before the patients receive medical treatment or get to the hospital [37].

Concerning the lagged effects, a relatively sharp decline in the OR pattern was observed over the lag days: indeed, the estimated ORs became lower than 1 at lag 5 or 6 (Table 4). This may be due to a phenomenon known as “harvesting effect”. This phenomenon occurs when a stressor mainly affects a pool of frail individuals, and, as a consequence, the size of this pool of susceptible subjects decreases in the subsequent days after exposure [27]. Indeed, this effect would be more apparent in subjects with pre-existent diseases (Figure S4). Similar trends were also found in previously published studies [12,39].

### 4.2. Subanalyses

We found larger ORs for PM_10_ (1 km) in the sub-period 2013–2015. This might be ascribed to the smaller sample size, as well as to the older age of the hospitalized subjects (Table S2), or to the higher cross-validated accuracy of PM_10_ estimates in these years (R^2^ = 0.84 in 2013–2015 vs. R^2^ = 0.75 in 2011–2015).

There was a trend to get a larger effect of PM in the urban area and of NO_2_ in the suburban area. In a previous US study (2002–2006) carried out in the elderly (≥65 years), the PM_2.5_ effects on CVD admissions were larger in urban counties rather than in nonurban ones, as in our study [40]. On the contrary, in another US study [4], larger effects of PM_2.5_ were found in rural areas with respect to urban ones. The inconsistency of these findings may be related to differences in exposure factors (e.g., sources of air pollution) or differences in health factors (e.g., lifestyles and access to close-by health care) in the analyzed population [4]. Thus, it would be worthwhile for future studies to consider the chemical composition of PM_10_, which may vary over time and different areas of residence, as a function of the emission sources [11].

Trends to higher risks were found in the elderly (≥85 years), in line with several other studies [8,10,21,41,42,43], likely due to the decreased physiological, metabolic and compensatory processes in these frail individuals [44].

Concerning the possible different risk by gender, our results are in line with some of the previous studies that found a higher risk in males [8,9,41]. Nevertheless, evidence of effect modification by gender remains uncertain. The range of plausible explanations is very broad, including either sex-related biological factors (lung volume, deposition, reactivity, and hormonal influences on chemical transport and systemic regulation) or confounding factors such as smoking habits and occupational exposures [45].

After stratifying by smoking status and occupational exposure, there were trends to larger effects of PM exposure in ever smokers and in those reporting occupational exposure. Indeed, studies on air pollution and smoking consistently demonstrated cardiovascular injury at levels and durations of exposure much smaller than those associated with lung cancer or even respiratory disease [46]. Furthermore, working exposure was linked to markers of adverse cardiovascular health, such as increased blood pressure, arterial stiffness and decreased heart rate variability [47]. Thus, the co-exposure to fumes/gases in the work environment may enhance the negative effect of air pollution on the risk of CVD acute events.

At last, the effect of PM exposure was larger in subjects with pre-existing cardiovascular/respiratory diseases. Indeed, there is evidence that coexisting chronic lung, heart or circulatory conditions in elderly populations may worsen as a consequence of the exposure to environmental pollutants [48].

### 4.3. Strengths and Limitations

The main strength of our study is to have combined previous analytical epidemiological data with routinely collected health statistics and mean concentrations of air pollutants estimated for each residential address, at high spatial resolutions. This provides new insights on acute cardiovascular effects of air pollution in small urban/suburban areas, where epidemiological evidence is somewhat limited due to the small number of monitoring stations [4]. Moreover, the pre-existing individual data gathered through questionnaires allowed us to stratify the models according to potential markers of different susceptibility.

Some study limitations should be acknowledged. The number of cases recorded in our general population sample was low, possibly leading to less precise and slightly overestimated effects, especially in the sub-period 2013–2015. Another potential limitation is to have not assessed individual exposures related to population mobility, transportation patterns, daily commuting, time spent outdoor and any other factor that may potentially contribute towards defining the “total human exposure”. In this regard, in previous surveys on indoor exposure assessment, data about the daily activity pattern in Pisa and in Po-Delta (North Italy) were collected in general population sub-samples [49]. Subjects spent most of their day indoors (about 85% in winter and 75% in summer). In particular, most of the time was passed at home, especially by subjects aged ≥65 years (75% in winter and 66% in summer). Indeed, our study population is mainly composed of elderly subjects (mean age about 80 years). Thus, we can reasonably assume that estimated exposure concentrations at the resolutions of 1 km and 200 m adequately represent the actual individual exposure. Future studies might benefit from the integration of various sources of exposures to provide a thorough overview of the effects of air pollution on CVD hospitalizations.

## 5. Conclusions

Combining analytical and routine epidemiological data with high-resolution pollutant estimates provides new insights into acute cardiovascular effects in small urban/suburban areas characterized by pollutant concentrations below the current EU regulatory limits, emphasizing that a “safe” exposure level may be difficult to establish. Moreover, targeted preventive measures should be planned at a population level to protect subjects at higher risk of detrimental effects.

## Figures and Tables

**Table 1:** Distribution of the 137 hospitalizations for acute cardiovascular events.

Hospitalization Characteristics	No. (%)
Disease group (ICD-9 code)	
Hypertensive disease (401–405)	1 (1)
Ischemic heart disease (410–414)	32 (23)
Diseases of pulmonary circulation (415–417)	9 (7)
Other forms of heart disease (420–429)	55 (40)
Cerebrovascular disease (430–438)	27 (20)
Diseases of arteries, arterioles, and capillaries (440–449)	8 (6)
Diseases of veins and lymphatics, and other diseases of circulatory system (451–459)	5 (4)
Year	
2011	30 (22)
2012	38 (28)
2013	26 (19)
2014	16 (12)
2015	27 (20)
Area of residence	
Urban (Pisa)	55 (40)
Suburban (Cascina)	82 (60)
Age class	
<85 years	87 (64)
≥85 years	50 (36)
Gender	
Female	69 (50)
Male	68 (50)
Smoking status	
Non-smoker	67 (49)
Ever smoker	70 (51)
Occupational exposure	
Not exposed	73 (53)
Exposed	64 (47)
Pre-existent cardiovascular/respiratory diseases	
No	77 (56)
Yes	60 (44)

**Table 2:** Mean, SD, median, 25th and 75th percentile, and interquartile range (IQR) of the estimated pollutant levels (µg/m^3^) throughout all the study days, for the whole period (2011–2015) and for the sub-period (2013–2015).

Pollutants	Mean	SD	Median	25th Percentile	75th Percentile	IQR
**2011–2015 (n = 411 ^1^)**						
PM_10_, 1 km	26.2	11.7	23.3	18.3	30.1	11.8
**2013–2015 (n = 207 ^2^)**						
PM_10_, 1 km	25.9	13.0	22.2	16.9	29.7	12.8
PM_2.5_, 1 km	17.4	11.3	13.0	10.2	19.5	9.3
PM_10_, 200 m	24.9	11.0	22.1	18.1	26.9	8.8
PM_2.5_, 200 m	16.0	9.2	12.9	10.2	17.5	7.3
NO_2_, 200 m	26.7	10.9	24.4	18.5	32.4	13.9
O_3_, 200 m	47.1	19.3	48.5	32.5	62.7	30.2

^1^ Three study days for each of the 137 hospitalizations. ^2^ Three study days for each of the 69 hospitalizations.

**Table 3:** Mean (SD) estimated pollutant concentrations (µg/m^3^) on the case days (day 0) and on the control days (day −7 and day +7), for the whole period (2011–2015) and for the sub-period (2013–2015).

Pollutants	Day 0	Day −7	Day +7	Δ(0,−7)	*p*-Value ^1^	Δ(0,+7)	*p*-Value ^1^
**2011–2015 (n = 137)**							
PM_10_, 1 km	27.6 (12.7)	25.9 (11.8)	25.5 (10.3)	1.7 (14.0)	0.149	2.1 (11.4)	**0.030**
**2013–2015 (n = 69)**							
PM_10_, 1 km	28.5 (14.8)	24.4 (12.3)	25.0 (11.5)	4.1 (15.8)	**0.033**	3.5 (12.0)	**0.021**
PM_2.5_, 1 km	18.9 (12.4)	16.8 (11.0)	16.7 (10.2)	2.1 (13.0)	0.181	2.2 (9.2)	*0.051*
PM_10_, 200 m	26.5 (11.3)	23.9 (11.7)	24.5 (9.9)	2.6 (11.8)	*0.073*	2.0 (9.0)	*0.070*
PM_2.5_, 200 m	17.1 (10.1)	15.7 (9.3)	15.6 (8.2)	1.4 (9.9)	0.231	1.5 (8.1)	0.123
NO_2_, 200 m	27.5 (11.3)	26.1 (10.8)	26.8 (10.8)	1.4 (6.8)	*0.096*	0.7 (6.0)	0.311
O_3_, 200 m	46.6 (19.4)	47.7 (19.7)	46.3 (19.1)	−1.1 (8.4)	0.269	0.3 (8.5)	0.781

^1^ Paired *t*-test. Significant *p*-values are in bold. Borderline significant *p*-values (<0.1) are in Italic.

**Table 4:** Acute effects of estimated pollution levels on the risk of cardiovascular hospitalizations: odds ratios (10 µg/m^3^ increase) and 95% confidence intervals through the conditional logistic regression models.

Lags	2011–2015 n = 137	2013–2015 n = 69					
	PM_10_, 1 km	PM_10_, 1 km	PM_2.5_, 1 km	PM_10_, 200 m	PM_2.5_, 200 m	NO_2_, 200 m	O_3_, 200 m
Lag 0	**1.137** **(1.023, 1.264)**	**1.268** **(1.085, 1.483)**	**1.273** **(1.053, 1.540)**	**1.365** **(1.103, 1.690)**	**1.264** **(1.006, 1.589)**	**1.477** **(1.058, 2.061)**	0.896 (0.710, 1.130)
Lag 1	**1.099** **(1.017, 1.188)**	**1.190** **(1.063, 1.332)**	**1.197** **(1.045, 1.372)**	**1.255** **(1.078, 1.461)**	**1.193** **(1.015, 1.401)**	**1.313** **(1.040, 1.658)**	0.930 (0.794, 1.089)
Lag 2	**1.062** **(1.006, 1.121)**	**1.116** **(1.036, 1.203)**	**1.126** **(1.029, 1.231)**	**1.154** **(1.049, 1.270)**	**1.125** **(1.016, 1.245)**	**1.167** **(1.012, 1.346)**	0.965 (0.885, 1.054)
Lag 3	1.027 (0.984, 1.071)	1.047 (0.990, 1.108)	1.059 (0.989, 1.133)	1.061 (0.998, 1.129)	1.061 (0.989, 1.139)	1.038 (0.947, 1.137)	1.002 (0.961, 1.046)
Lag 4	0.992 (0.942, 1.044)	0.982 (0.913, 1.058)	0.995 (0.908, 1.091)	0.976 (0.897, 1.061)	1.001 (0.905, 1.107)	0.922 (0.804, 1.058)	1.041 (0.954, 1.135)
Lag 5	0.959 (0.890, 1.033)	0.922 (0.825, 1.030)	0.936 (0.815, 1.075)	0.897 (0.782, 1.029)	0.945 (0.805, 1.109)	0.820 (0.654, 1.029)	1.081 (0.923, 1.265)
Lag 6	0.927 (0.837, 1.026)	0.865 (0.741, 1.009)	0.880 (0.726, 1.068)	0.825 (0.677, 1.006)	0.891 (0.710, 1.118)	0.729 (0.526, 1.011)	1.122 (0.890, 1.415)

Significant effects (1 not included in the confidence interval) are in bold.

## Data Availability

The data are not publicly available due to ethical restrictions.

## Supplementary Materials

The following are available online at https://www.mdpi.com/1660-4601/18/3/1164/s1, Table S1: Whole cohort characteristics (n = 1585). Table S2: Distribution of the 137 hospitalizations for acute cardiovascular events by sub-period. Table S3: Mean (SD) of estimated pollution levels (µg/m^3^) throughout all the study days (case and control days pooled), by year and area. Table S4: Acute effects of estimated pollution levels on the risk of cardiovascular hospitalizations: odds ratios (10 µg/m^3^ increase) and 95% confidence intervals through the conditional logistic regression models: time-stratified design. Table S5: Acute effects of observed (Passi monitor) pollution levels on the risk of cardiovascular hospitalizations: odds ratios (10 µg/m^3^ increase) and 95% confidence intervals through the conditional logistic regression models. Figure S1: Acute effects of estimated pollution levels (sub-period 2013–2015) on the risk of cardiovascular hospitalizations: log-odds ratios and 95% confidence intervals for the conditional logistic regression models with distributed lags, stratified by gender. Significant *p*-values for interactions are in bold. Figure S2: Acute effects of estimated pollution levels (sub-period 2013–2015) on the risk of cardiovascular hospitalizations: log-odds ratios and 95% confidence intervals for the conditional logistic regression models with distributed lags, stratified by smoking status. *p*-values for interactions are superimposed. Figure S3: Acute effects of estimated pollution levels (sub-period 2013–2015) on the risk of cardiovascular hospitalizations: log-odds ratios and 95% confidence intervals for the conditional logistic regression models with distributed lags, stratified by occupational exposure. *p*-values for interactions are superimposed. Figure S4: Acute effects of estimated pollution levels (sub-period 2013–2015) on the risk of cardiovascular hospitalizations: log-odds ratios and 95% confidence intervals for the conditional logistic regression models with distributed lags, stratified by cardiovascular/respiratory disease pre-existence. Significant *p*-values for interactions are in bold.
