# Supplementary material for: Short-Term Effects of Air Pollution on Cardiovascular Hospitalizations in the Pisan Longitudinal Study
Source: Int J Environ Res Public Health. 2021 Jan 28;18(3):1164. doi: 10.3390/ijerph18031164 (PMC7908381; doi:10.3390/ijerph18031164)
Supplement: Supplementary file 1 [file ijerph-18-01164-s001.pdf]

Table S1. Whole cohort characteristics (n = 1585).

Subject Characteristics	No. (%)
Area of residence	
Urban (Pisa)	645 (41)
Suburban (Cascina)	940 (59)
Age class	
<85 years	1532 (97)
≥85 years	53 (3)
Gender	
Female	831 (52)
Male	754 (48)
Smoking status	
Non smoker	740 (47)
Ever smoker	845 (53)
Occupational exposure	
Not exposed	882 (56)
Exposed	703 (44)
Pre-existent cardiovascular/respiratory diseases	
No	1330 (84)
Yes	255 (16)

Table S2. Distribution of the 137 hospitalizations for acute cardiovascular events by sub-period.

Hospitalization Characteristics	2011–2012 n = 68	2013–2015 n = 69	*p*-Value [1]
Disease group (ICD-9 code)			0.440
Hypertensive disease (401–405)	0 (0)	1 (1)	
Ischemic heart disease (410–414)	20 (29)	12 (17)	
Diseases of pulmonary circulation (415–417)	5 (7)	4 (6)	
Other forms of heart disease (420–429)	27 (40)	28 (41)	
Cerebrovascular disease (430–438)	11 (16)	16 (23)	
Diseases of arteries, arterioles, and capillaries (440–449)	4 (6)	4 (6)	
Diseases of veins and lymphatics, and other diseases of circulatory system (451–459)	1 (1)	4 (6)	
Area of residence			1.000
Urban (Pisa)	27 (40)	28 (41)	
Suburban (Cascina)	41 (60)	41 (59)	
Age class			**0.025**
<85 years	50 (74)	37 (54)	
≥85 years	18 (26)	32 (46)	
Gender			1.000
Female	34 (50)	35 (51)	
Male	34 (50)	34 (49)	
Smoking status			0.796
Non smoker	32 (47)	35 (51)	
Ever smoker	36 (53)	34 (49)	
Occupational exposure			0.263
Not exposed	40 (59)	33 (48)	
Exposed	28 (41)	36 (52)	
Pre-existent cardiovascular/respiratory diseases			0.432
No	41 (60)	36 (52)	
Yes	27 (40)	33 (48)	

[1] *Chi-squared* test. Significant *p*-values are in bold. Data are expressed as No. (%).

Table S3. Mean (SD) of estimated pollution levels (μg/m^3) throughout all the study days (case and control days pooled), by year and area.

Pollutants	Pisa	Cascina
PM_{10}, 1 km		
2011	30.6 (11.0)	28.5 (12.6)
2012	22.5 (5.8)	25.3 (8.8)
2013	27.2 (16.4)	24.0 (15.0)
2014	24.3 (8.6)	26.2 (11.7)
2015	28.5 (11.7)	25.6 (11.5)
$PM_{2.5}$, 1 km		
2013	18.9 (14.8)	16.5 (12.5)
2014	15.5 (6.7)	17.9 (9.1)
2015	19.0 (10.0)	16.7 (10.3)
PM_{10}, 200 m		
2013	27.3 (14.7)	24.5 (12.2)
2014	22.9 (6.3)	23.9 (7.6)
2015	27.7 (11.0)	23.7 (9.9)
$PM_{2.5}$, 200 m		
2013	17.6 (12.0)	15.8 (9.5)
2014	14.2 (6.5)	15.6 (8.1)
2015	19.0 (9.6)	14.9 (7.8)
NO_2, 200 m		
2013	31.9 (13.9)	28.3 (10.8)
2014	23.8 (7.4)	25.0 (10.6)
2015	26.3 (8.2)	24.1 (9.4)
O_3, 200 m		
2013	49.0 (23.3)	50.3 (18.7)
2014	47.8 (12.6)	40.9 (20.4)
2015	37.3 (20.4)	49.6 (16.7)

Table S4. Acute effects of estimated pollution levels on the risk of cardiovascular hospitalizations: odds ratios (10 μg/m^3 increase) and 95% confidence intervals through the conditional logistic regression models: time-stratified design.

Lags	**2011–2015 n = 137**	**2013–2015 n = 69**					
	**PM_{10}, 1 km**	**PM_{10}, 1 km**	**$PM_{2.5}$, 1 km**	**PM_{10}, 200 m**	**$PM_{2.5}$, 200 m**	**NO_2, 200 m**	**O_3, 200 m**
Lag 0	**1.117 (1.013, 1.231)**	**1.227 (1.077, 1.398)**	**1.234 (1.053, 1.446)**	**1.320 (1.044, 1.526)**	**1.223 (1.002, 1.516)**	**1.410 (1.025, 1.939)**	0.989 (0.787, 1.244)
Lag 1	**1.090 (1.013, 1.173)**	**1.164 (1.057, 1.282)**	**1.175 (1.045, 1.322)**	**1.205 (1.038, 1.408)**	**1.142 (1.008, 1.304)**	**1.278 (1.014, 1.610)**	0.992 (0.840, 1.172)
Lag 2	**1.064 (1.009, 1.122)**	**1.104 (1.031, 1.182)**	**1.119 (1.029, 1.217)**	**1.113 (1.025, 1.208)**	**1.103 (1.005, 1.210)**	**1.158 (1.003, 1.348)**	0.995 (0.890, 1.111)
Lag 3	1.038 (0.995, 1.083)	1.047 (0.991, 1.107)	1.066 (0.994, 1.143)	1.062 (0.995, 1.134)	1.065 (0.988, 1.148)	1.049 (0.945, 1.164)	0.997 (0.922, 1.079)
Lag 4	1.013 (0.965, 1.063)	0.993 (0.929, 1.063)	1.015 (0.933, 1.105)	1.015 (0.940, 1.095)	1.028 (0.938, 1.127)	0.950 (0.837, 1.079)	1.000 (0.906, 1.103)
Lag 5	0.988 (0.925, 1.057)	0.942 (0.857, 1.036)	0.967 (0.859, 1.088)	0.969 (0.870, 1.079)	0.993 (0.871, 1.132)	0.861 (0.706, 1.050)	1.002 (0.863, 1.164)
Lag 6	0.965 (0.882, 1.055)	0.894 (0.786, 1.017)	0.921 (0.785, 1.080)	0.925 (0.799, 1.071)	0.959 (0.802, 1.146)	0.780 (0.587, 1.036)	1.005 (0.814, 1.241)

Significant effects (1 not included in the confidence interval) are in bold.

Table S5. Acute effects of observed (Passi monitor) pollution levels on the risk of cardiovascular hospitalizations: odds ratios (10 μg/m^3 increase) and 95% confidence intervals through the conditional logistic regression models.

Lags	2011–2015 n = 137	2013–2015 n = 69			
	PM_{10}, Monitor	PM_{10}, Monitor	$PM_{2.5}$, Monitor	NO_2, Monitor	O_3, Monitor
Lag 0	**1.113 (1.017, 1.217)**	**1.268 (1.094, 1.469)**	**1.202 (1.024, 1.411)**	1.183 (0.996, 1.406)	**0.855 (0.741, 0.987)**
Lag 1	**1.084 (1.014, 1.158)**	**1.195 (1.073, 1.331)**	**1.151 (1.025, 1.292)**	**1.139 (1.005, 1.291)**	**0.901 (0.816, 0.996)**
Lag 2	**1.055 (1.008, 1.105)**	**1.127 (1.047, 1.212)**	**1.102 (1.020, 1.191)**	**1.096 (1.007, 1.193)**	0.949 (0.894, 1.009)
Lag 3	1.028 (0.990, 1.066)	**1.062 (1.007, 1.120)**	1.056 (0.996, 1.119)	1.055 (0.988, 1.126)	1.000 (0.960, 1.042)
Lag 4	1.001 (0.956, 1.048)	1.001 (0.939, 1.067)	1.011 (0.938, 1.090)	1.015 (0.933, 1.105)	1.054 (0.990, 1.122)
Lag 5	0.975 (0.913, 1.041)	0.944 (0.858, 1.038)	0.968 (0.866, 1.083)	0.977 (0.863, 1.107)	**1.110 (1.002, 1.230)**
Lag 6	0.949 (0.868, 1.038)	0.890 (0.778, 1.017)	0.927 (0.793, 1.084)	0.941 (0.792, 1.118)	**1.169 (1.010, 1.353)**

Significant effects (1 not included in the confidence interval) are in bold.

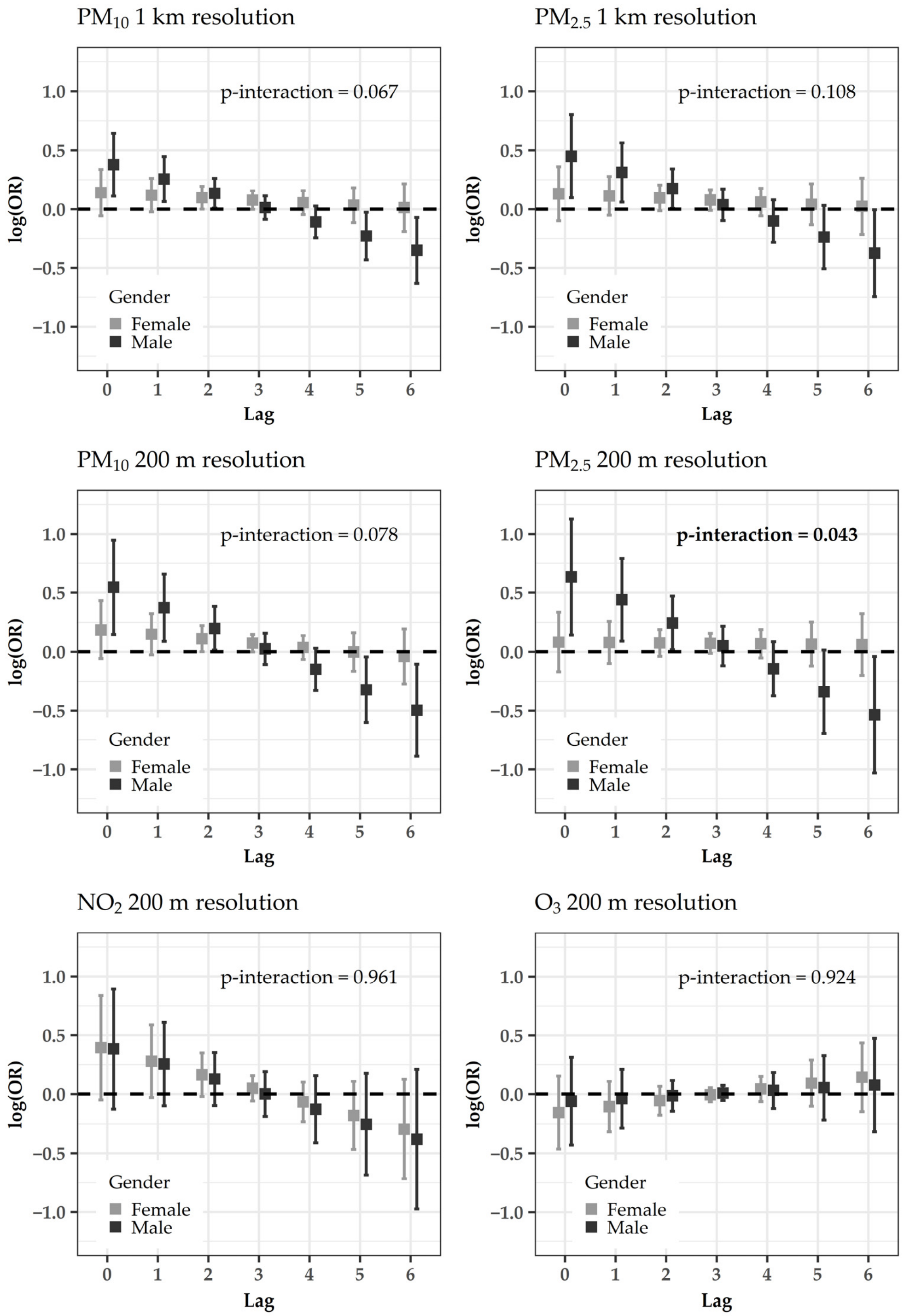

Figure S1. Acute effects of estimated pollution levels (sub-period 2013–2015) on the risk of cardiovascular hospitalizations: log-odds ratios (OR) and 95% confidence intervals for the conditional logistic regression models with distributed lags, stratified by gender. Significant *p*-values for interactions are in bold.

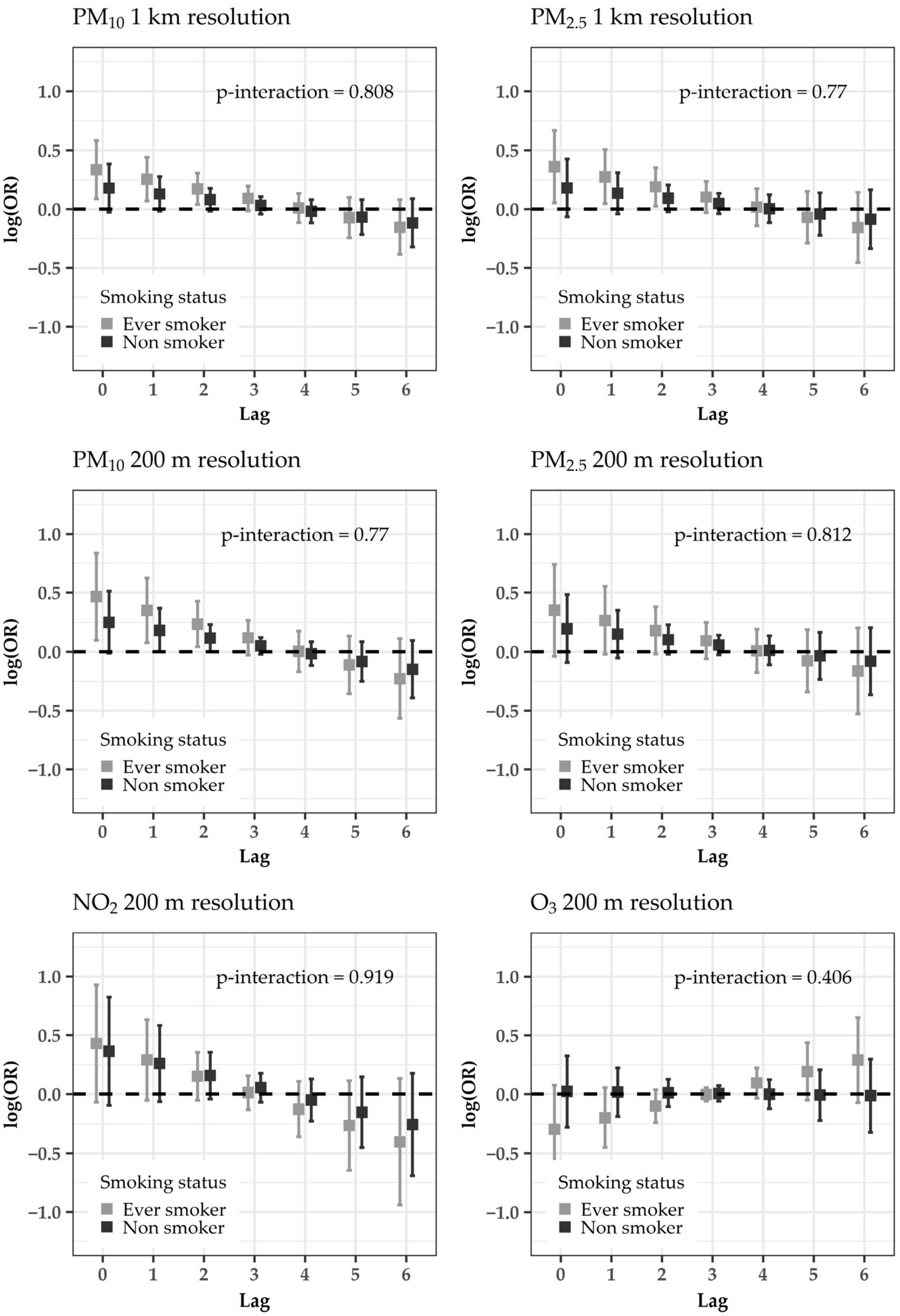

Figure S2. Acute effects of estimated pollution levels (sub-period 2013–2015) on the risk of cardiovascular hospitalizations: log-odds ratios (OR) and 95% confidence intervals for the conditional logistic regression models with distributed lags, stratified by smoking status. *p*-values for interactions are superimposed.

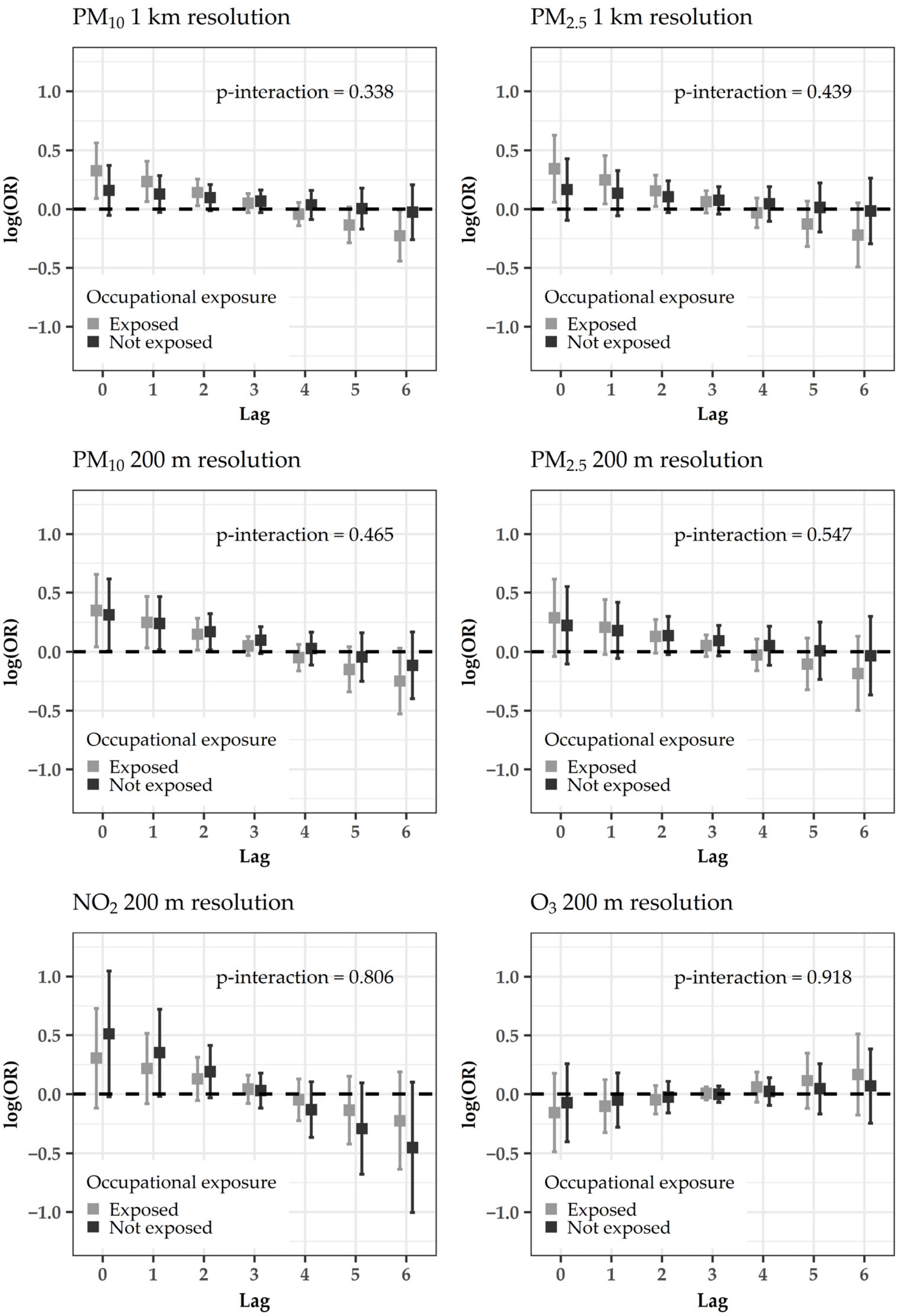

Figure S3. Acute effects of estimated pollution levels (sub-period 2013–2015) on the risk of cardiovascular hospitalizations: log-odds ratios (OR) and 95% confidence intervals for the conditional logistic regression models with distributed lags, stratified by occupational exposure. *p*-values for interactions are superimposed.

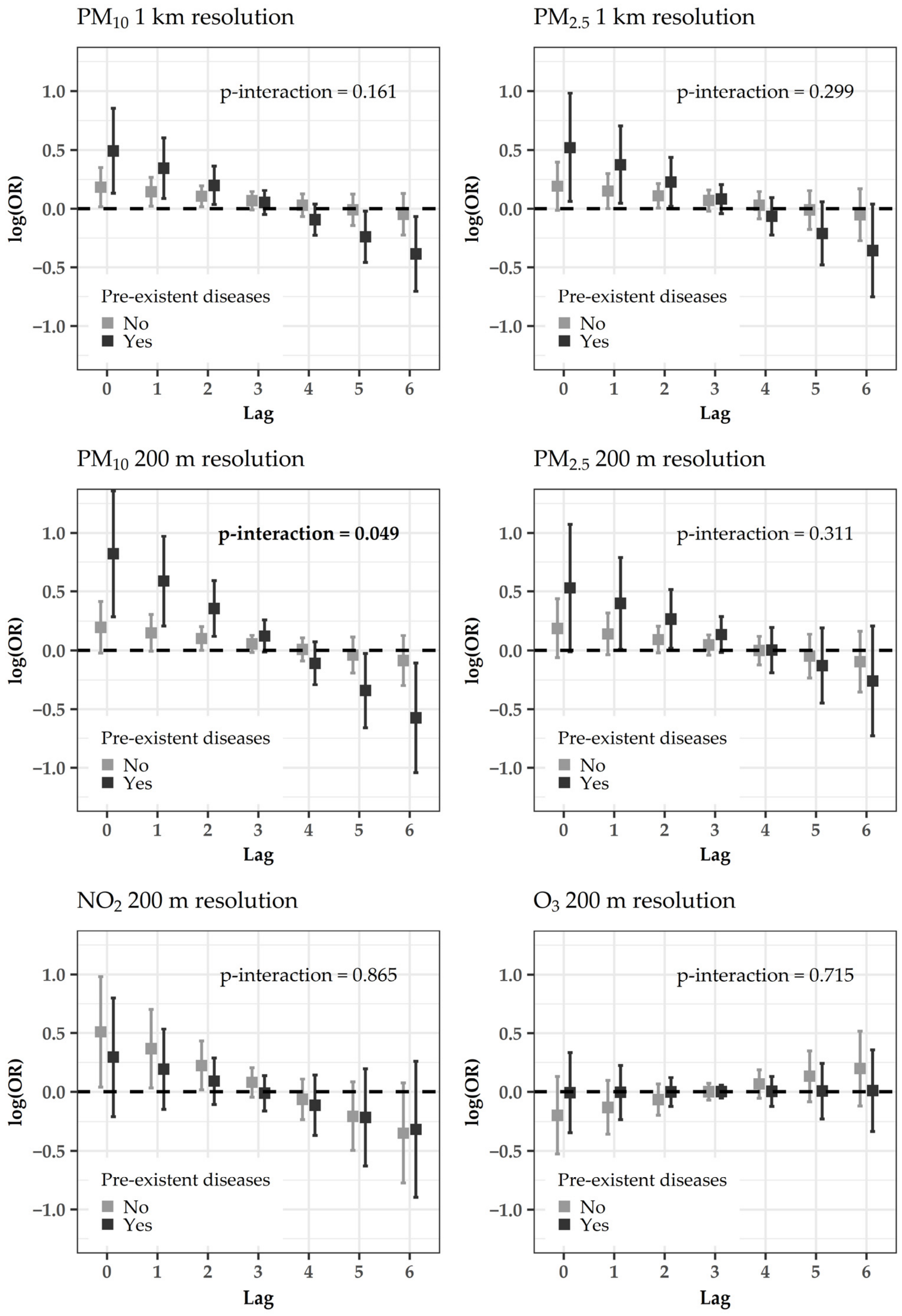

Figure S4. Acute effects of estimated pollution levels (sub-period 2013–2015) on the risk of cardiovascular hospitalizations: log-odds ratios (OR) and 95% confidence intervals for the conditional logistic regression models with distributed lags, stratified by cardiovascular/respiratory disease pre-existence. Significant *p*-values for interactions are in bold.
